# Scorpion envenomation in Brazil: Current scenario and perspectives for containing an increasing health problem

**DOI:** 10.1371/journal.pntd.0011069

**Published:** 2023-02-09

**Authors:** Clara Guerra-Duarte, Rafael Saavedra-Langer, Alessandra Matavel, Barbara B. R. Oliveira-Mendes, Carlos Chavez-Olortegui, Ana Luiza Bittencourt Paiva

**Affiliations:** 1 Diretoria de Pesquisa e Desenvolvimento, Fundação Ezequiel Dias, Belo Horizonte, Minas Gerais, Brazil; 2 Departamento de Bioquímica e Imunologia, ICB, Universidade Federal de Minas Gerais, Belo Horizonte, Minas Gerais, Brazil; 3 l’Institut du Thorax, Inserm UMR 1087/CNRS UMR 6291, Nantes, France; Muséum National d’Histoire Naturelle, FRANCE

## Abstract

Opportunistic scorpion species can colonize urban environments, establishing high-density communities that enhance the chances of human accidents. This scenario has been taking place in Brazil, in which some *Tityus* species have taken city centers, causing an explosion in the number of scorpion envenoming cases. The characteristics of this scorpionism epidemic in Brazil is discussed in the present work. The number of Brazilian scorpion stings has surpassed 120,000 cases in 2017, and has been maintained above this number ever since, representing a more than 3-fold increase in 10 years, which was higher than the number of cases for most of the neglected tropical diseases in the country. The escalation in scorpionism cases is even higher in some regions of Brazil. Fortunately, the proportion of mild cases has also increased in the analyzed period, as well as the number of victims seeking for medical attention within the first hour after the accident. The species *Tityus serrulatus*, *Tityus stigmurus*, *Tityus bahiensis*, and *Tityus obscurus* are traditionally accountable for most of the scorpion accidents in different regions of Brazil, but other species deserve to be closely watched. Despite scorpionism being a notable health problem in Brazil, accident prevention and pest control regarding this venomous animal have not been properly addressed by the scientific community nor by policy makers. Therefore, this review also aims to point possible fields of research that could help to contain the aggravation of the current scorpionism landscape in Brazil.

## Author summary

The presence of scorpions in Brazilian urban centers has been increasing over the last decade and has led to an important augmentation in the number of human accidents in this country. This review aimed to better understand this scorpionism epidemic in Brazil, analyzing the evolution and dynamics of the accidents per region, the victim’s profile, and the outcomes of the resulting reported cases. The main scorpion species related to these accidents were reviewed, but emerging threatening species are also pointed and discussed. Considering this current medical emergency, it would be expected to have more preventive measures being proposed by the political and scientific community, but this has not been observed. Therefore, this work also suggests different fields that could be important points of action to reduce and contain problems related to scorpionism. These recommendations ranges from improvements in housing and education in all levels to chemical, biological, and genetic forms of control of scorpion populations. The effort made in this review intended to call attention to the scorpion problem in Brazil and to be part of the solution.

## 1. Introduction

Scorpions are a very ancient group that originated as terrestrial animals approximately 300 million years ago and have persisted ever since [1]. They are widespread around the globe, present in all continents apart from Antarctica, and are adapted to a variety of environments, including high altitudes, deserts, rainforests, and caves [2]. Some scorpion species are endemic and dependent of their original habitats’ natural conditions, living in small populations with restrict mobility [3].

On the other hand, there are opportunistic scorpion species, capable of adapting and colonizing disturbed environments, living in high-density communities. This group can reproduce quickly [4] and can survive without food for long periods [5], which warrants them to thrive even amid unnatural conditions, like urban centers [6]. This life strategy causes the substitution of endemic species by opportunistic ones in disrupted or anthropized environments and can lead to increased chances of human encounters, resulting in harmful accidents [4,7–9]. This phenomenon occurs in several places in the world that are afflicted by scorpionism, such as North-Saharan Africa, Middle East, India, Mexico, and South America [10].

Brazil is comprised within these scorpionism’ hot spots. Casualties with these arachnids have been described in the country since 1915 [11], the same period in which antivenom therapy for scorpion stings was also developed locally [12]. Notwithstanding, scorpionism is likely to be occurring in the area long before that. It has been hypothesized that colonizing incursions in the country’s interior have disturbed the original habitat of some scorpion species, and the foundation of new towns created new exploring possibilities for these animals [6].

Despite the accumulated knowledge, along more than a century, on several aspects of scorpions, ranging from venom composition to ecology, scorpionism is not a solved issue in Brazil. A substantial annual increase in the number of reported incidents, surpassing the mark of 100,000 cases per year since 2017, has been occurring. No effective measures to contain this epidemic have been put in place, and, as a result, the number of accidents and deaths continues to grow. This constant increase in scorpion stings in Brazil raise concerns and demands a deeper understanding of the environmental, demographic, and socioeconomic factors associated, the implications of these incidents, the identification of the scorpion species, and how to better prevent them. This review aims to gather the available information concerning scorpionism in Brazil and proposes future directions to cope with this urban pest.

## 2. The problem: Scorpion envenomation in Brazil

To access epidemiology data of scorpionism in Brazil, the SINAN *(*Sistema de Informação de Agravos de Notificação) database from Brazilian Ministry of Health, which compiles health data of compulsory notification, was consulted. Data on reported scorpion accidents from 2007 to 2019 were evaluated. This time frame was chosen because the database system was modified in 2007 and the collected information pattern differed from the previous period (2000 to 2006) [13]. Data from 2020 and 2021 are still under revision. However, considering that 2020 was an atypical year due to the Coronavirus Disease 2019 (COVID-19) pandemic, we assumed it would be informative to access preliminary numbers on reported cases and deaths in this year. After 2020, scorpion accidents reported in Espirito Santo state are no longer available in SINAN database, as the state is reporting data on their own platform from thereon, which will compromise future analysis using SINAN data. SINAN was last updated in January 24, 2022.

Even though SINAN is a very important asset to study the epidemiology of scorpionism and other medical conditions in Brazil, inconsistencies in the available database concerning envenoming numbers have been found [14–16]. In addition, there is evident underreporting of scorpionism cases. Even in urban centers, it is estimated that as much as 10% of scorpion stings may not be reported to the official surveillance system [17]. Indeed, Tanajura and colleagues [18] detected that 4.35% of scorpionism cases that received medical attention in the state of Bahia were not reported to the national system. Therefore, the data presented in the following results must be critically analyzed, considering these restrictions.

**Fig 1:**
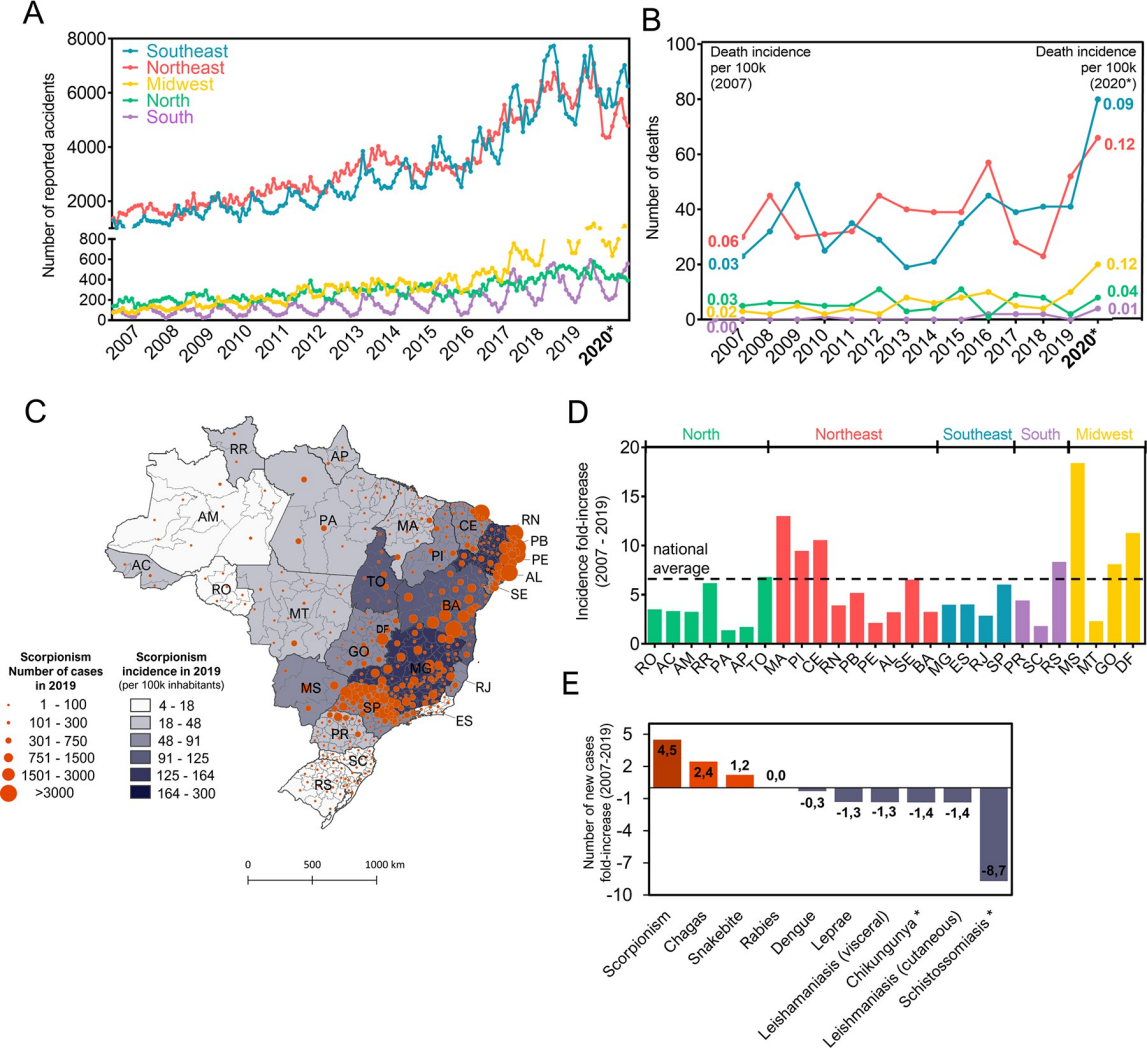
Reported scorpion accidents and related deaths in Brazil from 2007–2020*. (**A**) Number of reported scorpion accidents per month for each region. (**B**) Number of reported deaths related to scorpionism per year for each region. Death incidence per 100,000 inhabitants’ values for the years of 2007 and 2020 are written at the curves’ extremities. (**C**) Brazilian map showing scorpion incidence per federal unit in the year of 2019. Color intensities correspond to incidence rates, according to the legend. (**D**) Increase of incidence of scorpion accidents between the years of 2007 and 2019 per state. (**E**) Increase of incidence of neglected tropical diseases of compulsory notification between the years of 2007 and 2019 in Brazil. Graphs were produced using GraphPad Prism 9.0 software, and the map figure was produced using QGis software. The Brazilian map’s shapefile was downloaded from the Instituto Brasileiro de Geografia e Estatística–IBGE website (https://portaldemapas.ibge.gov.br/). Scorpion accidents and other neglected diseases numbers were recovered from the SINAN Database and Brazilian population numbers and estimatives, used for the incidence calculus, and were recovered from Brazilian Institute of Geography and Statistics (IBGE—Instituto Brasileiro de Geografia e Estatística).

Fig 1A shows the numbers of scorpion accidents monthly reported in each geographical region of Brazil, from 2007 to 2020. The number of notifications shows a constant increase along the years. The absolute number of accidents were substantially higher in the Southeast and Northeast regions since the beginning of the analyzed time-series, but all regions indicated a constant augmentation, notably in Midwest. The preliminary number of reported scorpion accidents in 2020 did not significantly increase but remained above 100,000 cases in this period. Considering that in 2020, due to the SARS-CoV-2 pandemic, people initially avoided seeking for hospital emergency care for conditions other than COVID-19, resulting in reductions of around 50% in emergency hospital attendance in Brazil [19,20], we hypothesize that the 2020’s accident numbers are likely to be highly underestimated.

Several studies in Brazil and in other countries have reported seasonality of scorpionism occurrence of [21–31]. According to the data presented in Fig 1A, scorpion accidents present a constant variation, tending to be more frequent in the warmer and rainy seasons. This marked seasonal variation behavior is well observed in the South, Midwest, and Southeast regions, but is less pronounced in Northeast and North, which may reflect the less evident seasons in these areas, as also acknowledged before in epidemiological studies concerning these regions [32–34]. Nevertheless, Monteiro and colleagues [17] observed that the Northern states, which are impacted by river floods in rainy season, have a positive correlation of this period with scorpion stings.

Despite the increase in the number of scorpion accidents, the number of deaths reported up to 2019 for each region did not present the same constant raising pattern, as seen in Fig 1B. However, in the year of 2020, when the number of reported scorpion accidents was lower than in 2019, the number of deaths seems to have skyrocketed. This could be an effect of envenomation complications, since people were avoiding going to emergency rooms due to the COVID-19 pandemic, and it has already been stated that envenoming outcome depends on the time in which medical treatment starts. We stress that numbers from 2020 are still under review, and notification errors could have occurred, as personally communicated by the technical group on venomous animals of Brazilian Ministry of Health, when consulted by email about these numbers. Nevertheless, until the date of this article’s submission (November 2022), numbers had not been updated, remaining as reported here. We calculated the death incidence per 100,000 inhabitants, per region, for the years of 2007 and 2020, using population numbers estimated by the Brazilian Institute of Geography and Statistics (IBGE—Instituto Brasileiro de Geografia e Estatística). The calculated incidence is shown by the numbers at the beginning and at the end of each curve in the graph (Fig 1B). If the real numbers are indeed within the reported range, death incidence per 100,000 habitants may have more than doubled for the regions with the highest number of cases and showed a 6-fold increase in the Midwest.

Fig 1C shows the Brazilian political map colored according to the scorpionism incidence per 100,000 inhabitants in 2019 for each Brazilian state, demonstrating high variability along the territory. Incidence ranged from approximately 4 cases/100,000 inhabitants in the state of Rio de Janeiro (RJ) to 300 cases/100,000 inhabitants in Alagoas (AL) and seems to be higher in the eastern part of Brazil. This map also highlights accidents’ hot spots, represented by the orange spheres. The sphere size is related to the absolute number of accidents occurring within a microregion. It is noteworthy that while in Southeastern states scorpion accidents are distributed more evenly through their territory, in Northeastern states, accidents are more concentrated in the state’s capitals, where most of the states’ population lives.

In face of this concerning rise in scorpionism, we can speculate on the current factors leading to it. According to IBGE (Brazilian Institute of Geography and Statistics) data [35], Brazilian population continues to grow every year. Therefore, it would be reasonable to assume that the increase observed in the number of scorpion stings could be following the population’s growth, and, therefore, the scorpion accident incidence would not change as much over the years. To address this possibility, we have calculated the rates of scorpion accidents incidence increase for each state, comparing the rates reported in 2007 and 2019 (Fig 1D). National average reveals that the number of reported scorpion accidents/100,000 inhabitants increased in all states, with a mean increase of 5.75-fold during this 13-year period, suggesting external factors leading scorpionism increase in the country. The scorpionism incidence in states that are traditionally inflicted by this condition, like Minas Gerais (MG), São Paulo (SP) (Southeast), and Bahia (BA) (Northeast), have grown below the national average range. Other states, despite not having high incidence rates in 2019, deserves attention due to the increase of more than 10-fold in the accident rate, which is the case of Maranhão (MA) and Ceará (CE), in Northeast, and Mato Grosso do Sul (MS) and the Federal District (DF), in Midwest. Also in this region, the state of Goiás (GO) presented a relevant increase, indicating that Midwest may be experiencing the effects of *Tityus serrulatus* colonization, as further discussed in the session below. The southernmost state of Rio Grande do Sul (RS), where scorpion accident reports increased more than 8-fold, is also a point of attention.

As reporting of scorpion accidents became mandatory in healthcare facilities only since 2010, the increasing number of reported cases could be simply a result of more notifications being processed, and not an increase in the number of accidents by itself. However, this reason alone may not explain the constant rising numbers, especially observed after 2017, as reported accidents with other venomous animals, such as snakes, did not follow the same pattern, with numbers fluctuating around 30,000 accidents per year throughout the analyzed period. Indeed, scorpion accidents is one of the only conditions of compulsory notification that present consistent growth along the years. When compared to other neglected tropical diseases notified to same national system in Brazil (SINAN), scorpionism is the condition with the highest raising trend and one with the highest absolute numbers of notifications (Fig 1E).

Since scorpions are affected by the environment, some studies have claimed that climate factors, such as global warming, could increase scorpion proliferation, maturation, and distribution [36–38], contributing to the observed accidents’ raise. Lacerda and colleagues [39] evaluated geographic and epidemiological characteristics of scorpion envenomation in São Paulo state and identified environmental factors that may be associated with these incidents. All the detected higher-risk areas presented lower precipitation, warmer temperatures, and a lower percentage of natural vegetation coverage, indicating a possible association of these factors with the occurrence of scorpion accidents. Interestingly, the percentage of urbanization was not different between the lower- and higher-risk areas, despite areas with less natural vegetation can be implied to be more urbanized [40]. More studies evaluating the correlation between rates of scorpionism, climate, and other environmental factors are important to predict future trends and to help to cope with the current epidemic [39,40]

**Fig 2:**
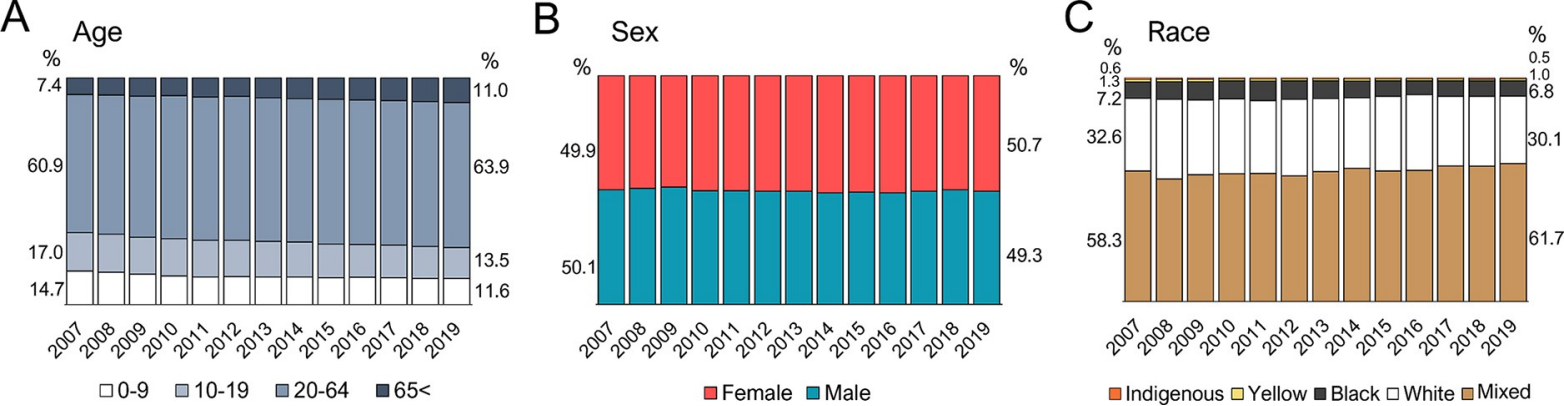
Demographic characteristics of scorpionism victims from 2007 to 2019 in Brazil. Bar graphs show the proportion of scorpionism victims along the years according to (**A**) age groups, (**B**) sex, and (**C**) auto-declared race. Numbers next to the first and the last bars represent the percentage of each analyzed group in the year of 2007 (left) and 2019 (right). Graphs were produced using Microsoft365 Excel software. Scorpion accidents’ information were recovered from the SINAN Database.

Considering the demographic characteristics of envenomed victims, the main profile has not change much between 2007 and 2019 (Fig 2). The proportion of accidents involving adults (20 to 64 years) and the elderly (>65 years) raised about 3%, whereas for the young population (10 to 19 years) and children (0 to 9 years), it has decreased at approximately the same rate (Fig 2A). These changes may reflect a demographic transition, with an observed aging of Brazilian population due to lower fertility and augmentation of life expectancy [41].

Opposed to what is observed for snakebite, scorpion accidents occurred in both sexes proportionally (Fig 2B). Snakebite is often associated with occupational risk, victimizing mostly agricultural workers, which are male in their majority [42]. As scorpion stings are more likely to occur within households or their surroundings [43], it has affected males and females indistinctively. However, when considering only the North region (S1 Table), the accident profile resembles those for snakebite, affecting more men in rural areas, as described previously [44–46]. On the other hand, women were more frequently stung by scorpions in Rio Grande do Norte (RN), in the Northeast region, which can be possibly related to scorpion urbanization, leading to incidents opportunities when performing housework, which is mostly a female activity in the state [32]. The larger prevalence of scorpion accidents in females was also noted for Bahia [47], Ceará [48], and Sergipe [49], which are also states from the Northeast region of Brazil. This divergency in scorpionism sex prevalence in Brazil demonstrates that assumptions made from combined data must be made carefully, when considering such a large and heterogenous country like Brazil.

According to the reported 2019’s National Household Sample Survey from the Brazilian Institute of Geography and Statistics (IBGE), it is estimated that 42.7% of Brazilians declare their race as White, 46.8% as Mixed (named as “*Pardos*” locally), 9.4% as Black, and 1.1% as Indigenous or Asian. The “mixed” population seems to be the most hardly hit by scorpionism, in a consistent tendency through the years, as it represents the majority (around 60%) of the scorpionism victims (Fig 2C). This higher incidence among the “mixed” population can be related to the higher social vulnerability of this group, which results in worst habitational conditions, lower access to water, sanitation, and proper waste management, leading to higher exposure to vectors, including scorpions [50]. It is important to underline that, even though the Indigenous population accounts for less than 1% of SINAN reported scorpion accidents, a study made with an indigenous community in the state of Acre (North) found that 14% of them had already been stung by scorpions at least once in their lifetime. This indicates that, although the indigenous group contributes with only a small proportion to the totality of scorpion accidents in Brazil, scorpionism is a relevant issue inside this community [51].

**Fig 3:**
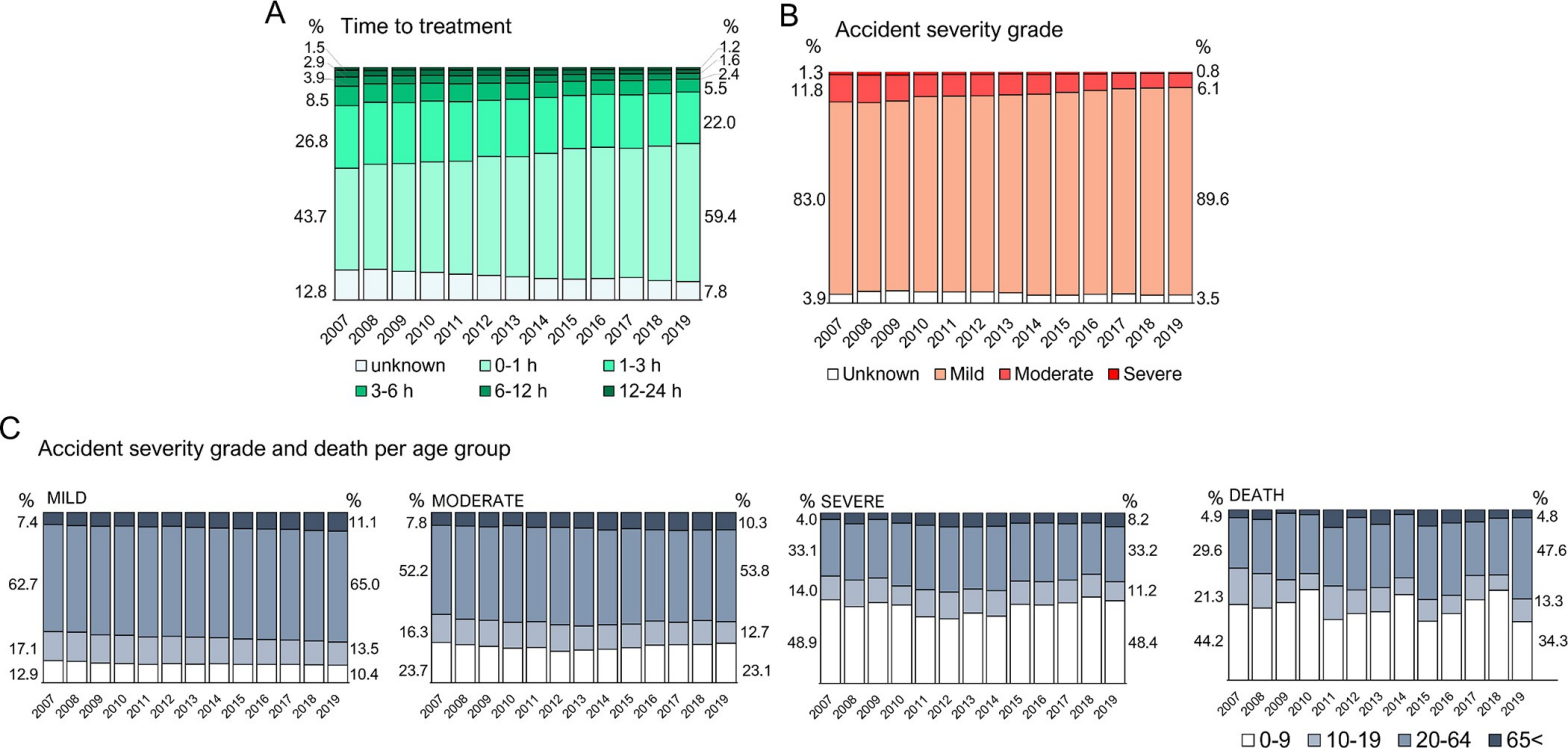
Medical aspects of scorpion accidents from 2007 to 2019. Bar graphs show the proportion of scorpionism victims along the years according to (**A**) the time elapsed from the accident until they searched for treatment, (**B**) the severity grade of envenoming, and (**C**) the age distribution of scorpionism victims according to accident severity grade and death. Numbers next to the first and the last bars represent the percentage of each analyzed group in the year of 2007 (left) and 2019 (right). Graphs were produced using Microsoft365 Excel software. Scorpion accidents’ information were recovered from the SINAN Database.

To further characterize the profile of scorpion accidents occurring in Brazil between 2007 and 2019, we accessed the time victims took to search for medical attention, as it is a fundamental parameter to ensure treatment success and prevent aggravation, and the severity grade of the accidents (Fig 3). The proportion of scorpionism victims searching for treatment within the first hour of envenoming has increased over the analyzed period, from 43.7% in 2007 to 59.4% in 2019 (Fig 3A), as already pointed by Chippaux in 2015 [52]. This shift is very positive, as the efficacy of the antivenom treatment is strongly related to the period in which it begins [53,54]. However, a proportional reduction in mortality due to this faster medical care intervention is not observed, but the proportion of mild cases has also increased.

Indeed, although the absolute numbers of accidents in all severity grades have raised unquestionably, the proportion of registered mild cases has increased from 83% to almost 90% of the total cases (Fig 3B). As a result, the percentage of moderate and severe cases were reduced in 2019 when compared to the first year of this time series. We can hypothesize that the substantial growth in the total number of registered scorpion envenoming cases may be due to more people searching for healthcare, even if it turns out to be mild envenoming, as a result of better information of the population about the conduct after a scorpion sting. On the other hand, the higher proportion of mild cases can reflect the faster medical care, as already mentioned. As people would look faster for the treatment, cases would not aggravate.

As it is well known that children are particularly vulnerable to scorpion venom [55], we analyzed case severity and deaths according to victim’s age (Fig 3C). The data show clearly that 0- to 9-year-olds are the group most vulnerable to aggravating envenoming, as this age group correspond to only about 10% to 13% of mild cases but represent almost half of severe cases, in a consistent pattern over the years. Lethality rates are also higher in this group as, although they account for less than 15% of total scorpionism cases, children under 9 represent more than a third of all fatal victims of scorpionism. It is relevant to stress that the proportion of adults in the total number of deaths in 2019, reaching almost half of all fatalities in this year, reveals that the life risk of scorpion envenoming cannot be disregarded in any age group.

Brazil is a country of continental dimensions and is very heterogenous in terms of climate, biomes, economic development, etc. Even inside a same state, broad variation in scorpionism incidence has been reported, as shown in Fig 1D [46,56]. More accurate identification of these scorpionism hot spots within Brazilian territory may be useful for allocating adequate resources for preventing and treating accidents, better reflecting the reality of the populations at greater risk [57]. Amado and colleagues [58] attempted to determine these vulnerable areas, considering not only accident incidence but also climatic niche modeling for all medically relevant species, investments in public health, accessibility to adequate treatment (hospital infrastructure and antivenom availability), and demographic data. This analysis put the North and Northeast regions as the higher priority for health investments. Nevertheless, the high absolute numbers of accidents in the Southeast and the elevated incidence growth observed in the Midwest and South regions contribute to the worrying picture installed in Brazil as a whole.

## 3. The culprits: *Tityus* scorpions

*Tityus* scorpions are undoubtedly the major responsible for the scorpionism epidemic that has been taking place in Brazil. It is likely that *Tityus serrulatus* is still the main culprit of severe cases, due to its apparent higher toxicity to humans [59]. This species is parthenogenetic and can colonize disturbed environments. These characteristics makes *T*. *serrulatus* scorpions capable of causing population “explosions,” forming large communities, which are almost impossible to eliminate [5,9], and it seems to be expanding its distribution. Other *Tityus* species also seem to be extending their geographical distribution as well, and their contribution to medically relevant cases have been increasingly documented. These facts highlight that, in the coming years, the scorpionism situation in Brazil can be further aggravated.

About 160 species of scorpions, from nine genera (*Tityus*, *Ananteris*, *Rhopalurus*, *Bothriurus*, *Thestylus*, *Ischnotelson*, *Jaguajir*, *Troglorhopalurus*, and *Brotheas*), belonging to four scorpion families (Bothriuridae, Buthidae, Chactidae, and Hemiscorpiidae) have been described so far in Brazil [60,61]. However, only Buthidae scorpions are considered of medical relevance and local accidents are caused mainly by scorpions belonging to *Tityus* genus. Other scorpion genera like *Rhopalurus* [62] and *Bothriurus* [63] can also be potential agents of human envenoming, but the lack of adequate information on scorpion accidents reports makes the actual contribution of these other species unknown.

**Fig 4:**
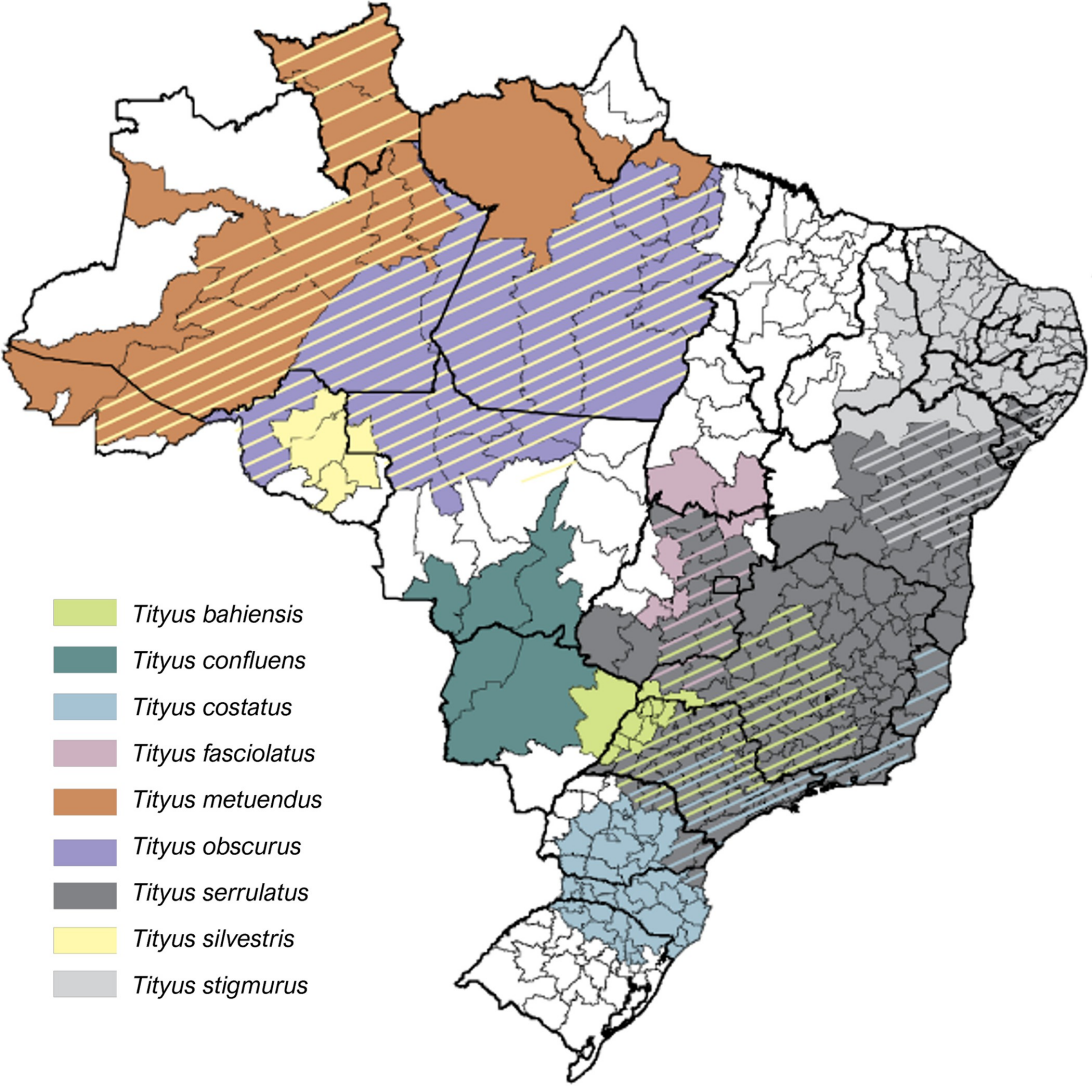
Distribution of the medically relevant *Tityus* scorpion species in Brazilian territory. Species’ distribution information was adapted from [69]. The map figure was produced using QGis software. The Brazilian map’s shapefile was downloaded from the Instituto Brasileiro de Geografia e Estatística–IBGE website (https://portaldemapas.ibge.gov.br/).

The genus *Tityus* has over 200 described species widely distributed throughout Central and South America, as well as in the Caribbean, and comprises the most medically important scorpion species across South America [64,65]. In Brazil, there are about 22 species described for the genus, but only four species are acknowledged as the main responsible for accidents: *Tityus serrulatus*, *Tityus stigmurus*, *Tityus bahiensis*, and *Tityus obscurus* [59,60,66–68]. Nevertheless, many recent reports are pointing to a more extensive contributions of other *Tityus* species to medically relevant cases. Fig 4 shows an estimated geographical distribution of the *Tityus* species more related to human envenoming in Brazil [69].

### 3.1. *Tityus serrulatus*

**Fig 5:**
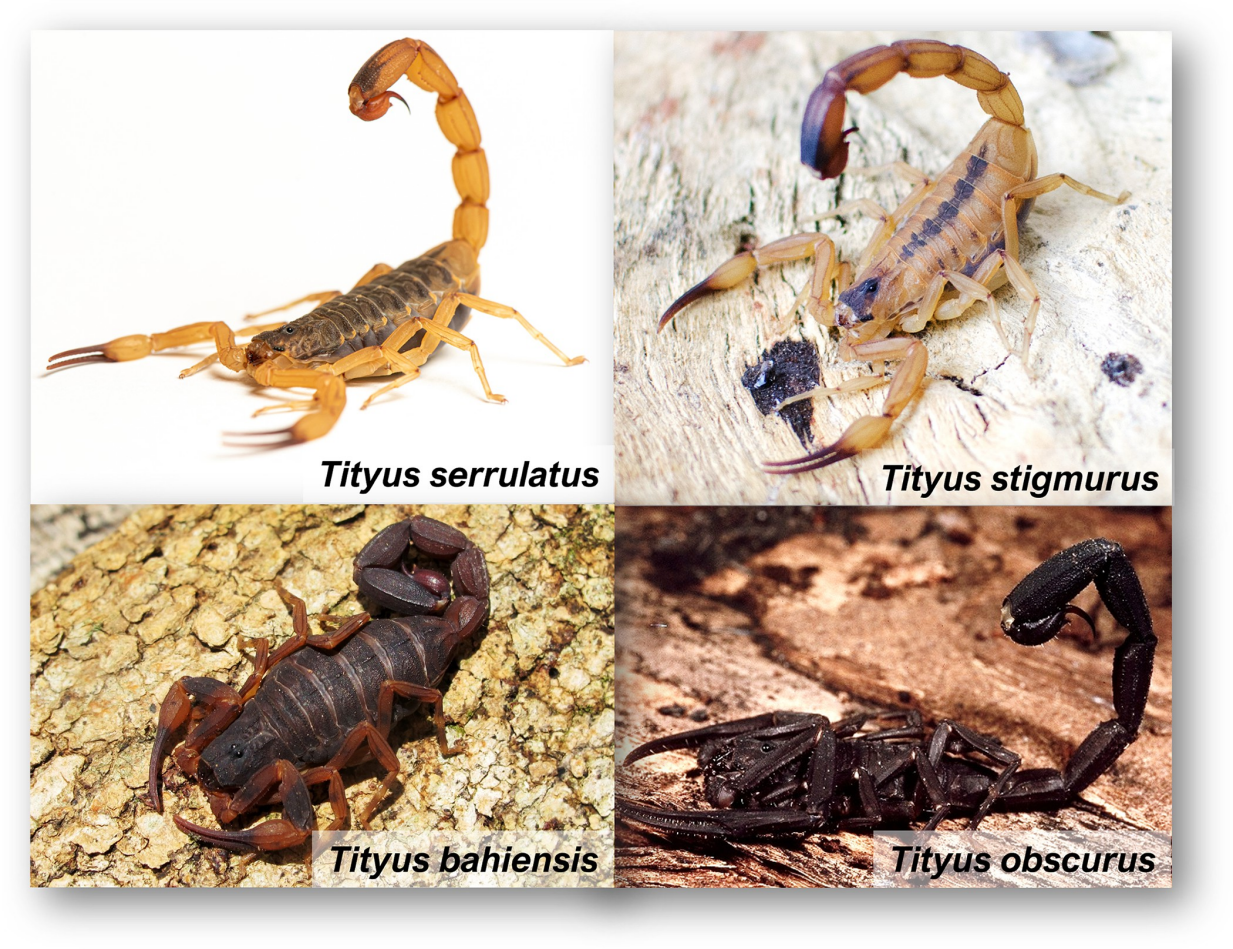
Main scorpion species of medical importance in Brazil. Image credits: Tityus serrulatus, Tityus stigmurus (Leonardo Noronha), Tityus bahiensis, and Tityus obscurus (Roberto Murta), provided under a CC BY 4.0 license.

Most of the severe scorpion envenoming cases in Brazil are caused by *T*. *serrulatus*, popularly known as “yellow scorpion.” Adult specimens typically measure 5 to 7 cm in length. As suggested by its common name, its coloration consists of pale yellow legs and pedipalps, with a darker shade of yellowish brown on the body and tip of the tail [70]. They display a serration along the dorsal face of the distal segments 3 and 4 of the tail, as small teeth, which confer the name “*serrulatus*” to the species [71] (Fig 5).

This species’ geographic distribution was previously restricted to Minas Gerais state (Southeast region), but due its easy adaptation to urban environments and its proliferation potential, *T*. *serrulatus* has expanded considerably over the Southeast, Northeast, South, and Central regions of Brazil, and its occurrence has been recorded in at least 19 of the 27 Brazilian states [4,72–76]. *T*. *serrulatus* has been also spotted in other countries like Ecuador and Argentina [77], where even a human accident occurred [78].

Its ability to reproduce by parthenogenesis is one of the factors that seems to contribute the most to *T*. *serrulatus* rapid proliferation and wide distribution [79]. A single specimen transported to a new location, finding the right conditions, can readily reproduce and develop a new colony [17]. As an example, Brazil’s political capital Brasília was originally devoid of *T*. *serrulatus*. But in a period of less than 20 years [77], the city has been invaded by this species. The spreading of *T*. *serrulatus* could have been supported by the road network, the main transportation system used in Brazil. In fact, some reports describe the introduction of this species in nonendemic states relating them to agricultural products transported from other states [70,72,74,80,81].

Pimenta and colleagues [5] reported that *T*. *serrulatus* individuals can survive up to 400 days without food, which contributes to the resistance needed to survive extensive displacement, even when resources are limited. Moreover, this study revealed that these scorpions were able to reproduce even after 209 days of food deprivation. Despite this species being usually considered an obligate parthenogenetic species, some isolated sexual populations with the presence of males were detected [82,83]. Nevertheless, nonparthenogenetic populations have a highly restricted geographic distribution [8,84], and it was observed that parthenogenetic reproduction in *T*. *serrulatus* also occurs in populations that still present sexual reproduction [85]. Even though parthenogenesis is likely to induce limited genetic diversity, intraspecific variations have been observed, which can also be a sign of adaptation to different environments by this species [77,86–88].

### 3.2. *Tityus stigmurus*

Another scorpion species of clinical relevance in Brazil is *T*. *stigmurus*, which is distributed predominantly in the northeastern region of Brazil [68]. They are 4.5 to 6 cm in length and are either golden-tan or yellowish-brown colored. They present a dark stripe over the mesosoma with either yellowish or orange pedipalps [68] (Fig 5).

The species have been reported in the states of Alagoas, Ceará, Paraíba, Piauí, Rio Grande do Norte, Sergipe, Bahia, Pernambuco [34,83], and on the island of Fernando de Noronha, probably introduced by transportation of goods from the continent [89]. Notably, *T*. *stigmurus* occurrence was already detected in urban areas in the Municipality of São Paulo (southeast region of Brazil) [90], suggesting this species can also be spreading across the country through road transportation and establishing new communities.

Like *T*. *serrulatus*, *T*. *stigmurus* is predominantly parthenogenetic, with few recorded sexual populations [79,91]. *T*. *stigmurus* also present a yellowish body color and may have a superimposed occurrence in the Cerrado and Atlantic Forest biomes with *T*. *serrulatus*, which makes it difficult to estimate the real contribution of each species to the epidemic scenario in some parts of the Northeast region, as both species are synanthropic and well adapted to urban centers [43,60,92]. Interestingly, it has been observed that *T*. *stigmurus* can be a more aggressive species when associated to others, being more effective in colonizing urban areas [93]. The predominance of *T*. *stigmurus* in the city center of Salvador compared to the presence of *T*. *serrulatus* in areas with reminiscent vegetation within the same region corroborates this observation and point that *T*. *stigmurus* may be indeed the main responsible for scorpion accidents in the Northeast region of Brazil, despite possible expansion of *T*. *serrulatus* in this area as well [92]. Reports of *T*. *stigmurus* envenoming point out to a severity alike the ones caused by *T*. *serrulatus*, with the exception of lethality, which seems higher in the later species [34].

In spite of that, confirmed deaths by *T*. *stigmurus* are being increasingly reported [22]. The venom of *T*. *stigmurus* can have a lower experimental median lethal dose (LD_50_) when compared to *T*. *serrulatus* and *T*. *bahiensis*, suggesting a higher toxicity of this venom [94]. However, as individual and populational differences in scorpion species venom composition have been reported [86–88,95], it is difficult to assess whether the reported difference in LD_50_ values directly reflects the venom toxicity of each species. Factors as the time after the last extraction of the venom, types of prey present in the diet, and different processing techniques can alter venom composition and toxicity [88,96,97]. Moreover, as the LD_50_ is estimated using mice, venom toxicity levels might be different in humans as previously reported for snake venom [98].

### 3.3. *Tityus bahiensis*

*Tityus bahiensis*, another medically relevant species in Brazil, was the very first Brazilian scorpion species to be described [4]. This species usually reaches 6 to 7 cm in length. Its cephalothorax and tergites are dark, with reddish brown at the top. They have light legs with black spots, pedipalp is usually light brown, being part of the tibia dark brown. Its telson is reddish, the pincers’ tips and the stinger are brown or black, which is a way of identifying the species [4,99] (Fig 5).

Interestingly, contrary to what the name “*bahiensis*” might suggest, the original range of distribution of this species does not include Bahia state. *T*. *bahiensis* geographical distribution includes Minas Gerais, Goiás, São Paulo, parts of Mato Grosso do Sul, and Paraná (Southeast, Midwest, and South regions of Brazil), as well as Argentina and Paraguay [4,73].

Unlike the species mentioned above, *T*. *bahiensis* exhibits sexual reproduction and brown body coloration. In general, accidents involving *T*. *bahiensis* are considered less severe than the ones caused by *T*. *serrulatus* [59,100,101]. *T*. *bahiensis* distribution seems to have shrunk over the years, being consistently substituted by *T*. *serrulatus* [7]. Nevertheless, *T*. *bahiensis* is still an opportunistic species capable of colonizing urban zones, with fewer specific ecological requirements to survive when compared to other *Tityus* species, but with less plasticity than *T*. *serrulatus* and *T*. *stigmurus* [102].

### 3.4. *Tityus obscurus*

*Tityus obscurus*, known as the Amazonian black scorpion, is the one of the main agents of scorpionism in Northern Brazil and one of the largest *Tityus* species, growing up to 6.5 to 10 cm. It is characterized by its black color, flattened body and legs, and relatively thin claws. Juveniles have a brown body and appendix, dark stained [99] (Fig 5).

*T. obscurus* is a senior synonym of *Tityus paraensis*, Kraepelin, 1896 and *Tityus cambridgei*, Pocock, 1897 [103], and the names *T*. *cambridgei* and *T*. *obscurus* have been used indistinctly [17]. This species has been reported only in the region of Brazilian Amazon, which comprises Amazonas, Mato Grosso, Pará, and Amapá states [60,104]. It is well adapted to the high temperatures and humidity typical of the rain forest, which also extends the distribution of *T*. *obscurus* to French Guyana, Suriname, Ecuador, and Venezuela [103]. None of the other abovementioned congeneric species have been reported in this biome.

This species presents sexual reproduction. The venom of *T*. *obscurus* was reported as less toxic when compared with Brazilian scorpions’ venoms of medical relevance, especially *T*. *serrulatus* [94]. Nevertheless, *T*. *obscurus* venom can induce similar symptoms and lethality to mice but at higher dosage and different time frame [105]. Interestingly, *T*. *obscurus* seems to contain at least two morphologically similar but toxinologically distinct subspecies, since different neurological manifestations have been observed in envenomation attributed to *T*. *obscurus* in the western area of Para state, such as symptoms described as “electrical shock,” which causes body muscular contraction [33,104,106]. Torrez and colleagues [107] reported neurological disorders and electric shock-like sensations in 97% and 89%, respectively, of patients stung by *T*. *obscurus*. Despite these exuberant symptoms that may be present in *T*. *obscurus* envenoming cases, most of the accidents with this species are reported as mild [33]. Nonetheless, there has been a notable increase of the severity of systemic manifestations in the cases reported for *T*. *obscurus* stings more recently [17].

### 3.5. Other *Tityus* species of medical relevance

Although the four above-described species have been historically classified as the main responsible for scorpion accidents in Brazil, the offending species is almost never identified in accidents’ reports, making it impossible to acknowledge the exact contribution of each species to the epidemiological scenario. Recent scientific publications have described relevant human accidents with other scorpion species, especially in the Amazon region, which has the highest *Tityus* species diversity [17]. Besides *T*. *obscurus*, the species *Tityus metuendus*, *Tityus silvestri*s, *Tityus bastosi*, *Tityus apiacas*, and *Tityus strandi* seem to be responsible for most envenomation cases in Brazilian Amazonia [106,108].

Monteiro and colleagues [109] published a case report in Manaus (Amazonas) of a *T*. *silvestris* severe accident in a 39-year-old man that required intensive care. Despite antivenom treatment, the patient still presented muscle spasms for three days after the accident. Confirming the importance of *T*. *silvestris*, Coelho and colleagues [110] reviewed a series of 13 cases of scorpionism with this species in Pará state, in which three patients showed systemic symptoms. In 2017, Silva and colleagues [111] presented a 4-case series of scorpion envenoming in the South of Amazonas state, in which the offending species was identified as *T*. *apiacas*. The symptoms of these patients resembled the ones caused by *T*. *obscurus*, with “electric shock sensation” being reported. Gomes and colleagues [45] reviewed 151 scorpionism cases, also in the Amazonian region, describing *T*. *metuendus* as the main causing agent (68% of the cases), but other 6 species were also involved (*T*. *silvestris*, *T*. *raquelae*, *T*. *apiacas*, *T*. *dinizi*, *Brotheas amazonicus*, and *Ananteris dekeyseri*). Most of these reported cases were mild, but severe symptoms were observed and required ICU admission of five patients (four for *T*. *metuendus* and one for *T*. *silvestris*). As deforestation accelerates, these Amazonian *Tityus* species may have had their habitats disturbed, being dislocated. Considering this panorama, human encounters with these scorpions are more likely to occur, even though they have not been described earlier as opportunistic species [102].

*Tityus costatus* [112] and *Tityus fasciolatus* [113] are species present in the Midwest and Coastal regions in Southeast and South regions of Brazil, more likely to be equilibrium species, living in less dense communities and stable environments [102]. In spite of this, they also are capable of causing relevant human accidents. The LD_50_ of *T*. *fasciolatus* venom indicates that it can be more toxic than *T*. *obscurus* [114], and *T*. *costatus* has been responsible for recent city infestations, with multiple individuals being found inside dwellings, as it has been reported in southern cities of Brazil in the past few years [115]. Interestingly, *T*. *costatus* may have been the agent of the first scorpion accident reported in Brazil [4]. As for the Amazonian species described above, the actual contribution of *T*. *costatus* and *T*. *fasciolatus* to the number of accidents in Brazil remains unknown, as their venom composition, envenoming symptoms, and response to current antivenoms also do. In addition, *T*. *pusillus* [116] and *T*. *brazilae* [117] have also been reported as agents of sparce human envenomation in Northeast region. Initially a species of wide distribution in dry areas but collected only seldom [118], *T*. *confluens* has been spotted more frequently in the past few years, especially in the Midwest, where the number of accidents is significantly growing in the past few years. As this species has been responsible for human deaths in Argentina [119] and has been expanding its distribution, penetrating in urban environments such as the city of Buenos Aires [120], *T*. *confluens* must be more closely surveilled and regarded as a potential threat in Brazil.

## 4. The possible solution

In Brazil, many *Tityus* species have now dominated several urban centers, and other opportunistic species may follow this trend. Within cities, scorpions find abundant resources for their living, such as food and shelter. Due to their efficient life strategies, together with the fast and unplanned occupation of urban space, climate change, and environmental degradation currently going on, it may be an unrealistic goal to eliminate scorpions from cities. Nevertheless, prevention and pest management are essential to stop the continuous increase of scorpion accidents.

### 4.1. Accident prevention

Preventive measures recommended by Brazilian Ministry of Health involves maintaining households and peridomestic areas clean, avoiding pilling up waste; preventing the entry of scorpions in houses by sealing doors, windows, drains, and walls; inspecting shoes and clothing prior to dressing; eliminating possible scorpion preys, such as cockroaches; and presenting natural scorpion predators such as night birds, lizards, and frogs [121]. As scorpions are unable to climb smooth surfaces, using materials with this characteristic to cover walls and furniture may also help to avoid accidents [122]. However, taking the social economics reality of Brazil, a significant part of the population may be unable to follow these prevention recommendations, as proper living conditions, adequate water supply, sanitation, waste collection, and housing are not accessible to all.

Housing improvement has been acknowledged as an important tool for vector control in urban areas. Openings in plumbing, loose-fit doors and windows, and foundation cracks can favor scorpion’s entering in households; therefore, scorpionism control can benefit from house construction improvements [123]. Although it is not a simple measure to be taken, being associated with high costs and long-term interventions, improving dwellings construction, and providing reliable piped water supply are likely to impact not only the scorpionism matter but also other several aspects of human health [124].

The preventive measures recommended by the Ministry of Health have been acknowledged as important since the 1920s [12], when Brazilian Researcher Ezequiel Dias, after who one of the Brazilian antivenom producer labs was named, reported several strategies to fight scorpions in the state of Minas Gerais. Given that these recommendations are common sense and of undeniable importance, the skyrocketing numbers of scorpion accidents in Brazil in the last years points that they are either not enough for preventing accidents or not correctly followed by the population. It would be expected that new initiatives to tackle this issue would be proposed by health authorities at some administrative level, but we were unable to identify any of such measures.

To illustrate, Spirandeli-Cruz and colleagues [125] proposed a very complete program for scorpion control in the city of Aparecida, in São Paulo state, in the 90s. The authors also highlighted the importance of having antivenom availability in the city. Nevertheless, after an initial benefit, the trend in scorpion accidents in this city has risen, as reported to SINAN in recent years for Sao Paulo state, apparently disregarding the measures proposed in the 90s. In 2019, a 1-year-old girl died in this city after being stung by a scorpion and not receiving prompt treatment, as antivenom was lacking in the city hospital, indicating the discontinuity of the proposed measures.

It is noteworthy that there is shortfall of information and education concerning prevention of envenoming in Brazil. The problem starts at school, where children are not properly taught how to deal with this common threat. Failure in providing adequate information regarding venomous animals are commonly found in schoolbooks, and this has been systematically reported [126]. Informing the population would be a valuable tool to empower citizens to prevent accidents [32]. A study in Rio de Janeiro state showed that ophidic accidents correlated with illiteracy, indicating that educational level may have contributed to vulnerability to accidents with venomous animals [127]. The educational problem extends beyond the basic level, and inadequacy of proper training in the formation of health professionals is also evident [15]. The few initiatives in proposing courses in this area suffer from low adherence. This impacts not only clinical management, leading to antivenom misuse, but also favors underreporting of cases as diagnosis is not always evident [128]. Notwithstanding, school-based interventions can be effective in promoting behavioral changes toward adopting preventive measures against urban pests [129,130]. Communication through popular media channels could also help to inform the general population in a more massive way [39], and it is remarkable that, in the period of higher incidence of scorpionism, accidents and related deaths are frequently present in newspapers and in the television. However, these media channels could be more intensively used to educate the population about prevention.

As acknowledged by the World Health Organization (WHO), social mobilization and community engagement are essential for effective vector control strategies [131], commonly adopted in programs aiming to tackle mosquito-borne diseases [132]. Even though scorpions and other arachnids were not included in this mentioned WHO initiative, scorpion control shares many characteristics with mosquito control, as both are urban pests acting directly in households, and much can be learnt from what has been done in this field. It is a consensus that engaging communities is challenging and goes beyond simply providing adequate information. Involving stakeholders in actions and decisions and knowing the sociopolitical, economical, and cultural context of the approached communities to stablish dialogues are necessary to design successful plans to achieve population participation [133].

Monitoring areas with high incidence of scorpionism can help to shape public policies. However, having the adequate professional human resources to cover the city’s territory can be a drawback. In this sense, citizen science, an approach that engages the population in data collection, can be a potential solution that has already been tested for mosquito control [134,135]. For this to be successful, scientists and professionals must work closely and horizontally with the participants and improved access to internet and smartphones must be granted. Yet, a broader surveilled area with lower costs may be achieved using this strategy, in addition of improving scientific literacy and engagement of communities toward scorpion control [136].

### 4.2. Pest control

Control of the urban scorpion population size is essential to reduce human accidents, and it is regulated by legislations and recommendations by official organs in Brazil [99]. However, methodologies related to this has not been an issue broadly addressed by the scientific community, and no breakthroughs have been observed in this area in recent years. Scorpions can easily hide from traditional pest control measures, as they can find shelter, food, and water in drainage systems, making specimen collection, chemical and biological control approaches more difficult to be adequately employed.

Specimen collection is a common recommended practice to reduce the urban scorpion population. If well planned, considering scorpion incidence, this simple action can significantly reduce infestations. Scorpions bear a condition that favors their visualization, as they become fluorescent in ultraviolet (UV) light [137], due to components of their exoskeleton [138,139]. Indeed, Brites-Neto and colleagues [140] reported that nocturnal specimen collection using UV light increased in 114% the annual mean of collected individuals, when compared to daily collection expeditions in the city of Americana, in São Paulo state. The number of accidents also decreased after adopting this collection methodology in this city.

Glue traps have been proposed to capture *Loxosceles* spiders [141], alone or combined with inseticides [142], and it would be reasonable to presume that a similar approach could be applied to scorpions, but no reports on this were found in the literature. Traps using pheromone baits were also proposed successfully as a control measure to tick infestations [143,144]. This kind of trap coupled with a solar device able to electrocute lured ticks has also been recently reported as a viable and cost-effective alternative to tackle tick infestation [145]. However, very little is known about scorpions pherormone communication to make use of this approach. Nevertheless, the presence of pectens, sophisticated chemotactile organs in scorpions, points that this could be an important field of study that can be addressed for controlling scorpions’ infestation [146].

The use of chemical control of scorpion population is controversial among the scientific community. From one side, there are chemicals that seem to efficiently kill scorpions experimentally [147]. On the other hand, many used pesticides seem to irritate the animals and cause a dislodgement effect, as scorpions can detect pesticides through their pectens, close their pulmonary stigma, and move away from the irritating source, preventing the substance’s lethal action. This displacement stimulated by pesticides can contribute to human encounters in populated areas and, therefore, cause accidents. Considering this, in 1955, Bücherl proposed that, to be efficient, insecticides must be applied in all intended areas, at the same time, repeating the treatment for three times a month, for three months [148]. As a public policy, these conditions seem difficult to be followed, requiring highly coordinated logistics to be achieved. The adapted use of nonspecific insecticides, in addition of having low efficacy, are more prone to cause environmental contaminations, as they tend to be used in larger amounts [122].

It is curious that, considering the medical relevance of scorpions and the common infestation of this pest, there are not much research around finding more efficient pesticides for scorpions in laboratory trials nor addressing a fit strategy to apply it in the field. Ramires and colleagues wrote a very thorough book chapter on this matter, compiling not only articles but also conference papers and other communications, to gather information concerning arachnids’ chemical control [122]. Among the few published articles, Albuquerque and colleagues evaluated the effect of the pesticide cyhalothrin in a concentrated preparation sprayed on premises in Pernambuco by health agents, aiming to reduce *T*. *stigmurus* population [149]. The authors monitored 69 premises treated with this compound, seeking to report scorpion deaths and appearances. After the chemical compound application, scorpions were found in 42% of the locations, and only a minor part of the individuals were found dead. Authors concluded that the used method failed to kill scorpions and may have caused dispersion in the studied area. This work also applied a questionnaire for the treated houses’ residents, which revealed that this studied population showed little interest in the preventive measures presented by the health agents.

The work of Albuquerque only made one application of the tested compound. A field trial, reported by Ramsey and colleagues, had a more successful outcome when using repeated applications of different pyrethroid pesticides (cyfluthrin WP, bifenthrin WP, or deltamethrin SC) in the State of Morelos in Mexico, where a high scorpionism incidence had been reported [150]. After three applications of the different pyrethroids in a 3-year period, scorpion prevalence, house sightings, and envenoming cases were significantly reduced in the sprayed areas. Moreover, the used formulations did not seem to cause irritation in the animals. This approach, however, has not been tested in Brazil with *Tityus* scorpions, to verify if this would also be successful in the local context.

Santos and Albuquerque also tested a pyrethroid pesticide (Bifenthrin 20% w/v), in a pilot study also aiming to kill *T*. *stigmurus* [151]. The experiment was conducted in experimental conditions, using plastic recipients containing the individuals, and the effects caused by the substance were evaluated. The scorpions reacted to the pesticide, apparently sensing its presence. Some neurotoxic effects could be observed in the scorpions but seemed to be reversible in adults. For juveniles, the bifenthrin appeared to cause irritation. Therefore, this pyrethroid did not seem efficient as a pesticide for this scorpion species, and a good candidate to be used as a chemical control agent to *Tityus* scorpions is still lacking.

The biological control of the scorpion population is another measure that could be adopted, with the advantages of avoiding possible environmental contaminations and being mostly cheap. Several natural scorpion predators have been acknowledged, including other scorpions, arachnids, frogs, lizards, birds, and bats [152]. It is of popular knowledge in Brazil that chickens are good scorpion predators. This has been traditionally a common adopted strategy in rural settings, but, with the astonishing increase in the scorpion population in Brazil recently, citizens associations, urban communities, schools, and even city halls are also building urban henhouses as a strategy to cope with scorpion infestations. Indeed, Murayama and colleagues proved that hens are voracious scorpion predators and are also venom-resistant at some extent [153]. Although hens fit the criteria for being efficient predators to be used in biological control, it must be considered that their life habits did not match the scorpions’, which are nocturnal animals and stay usually hidden, while chickens pack in the open during the day.

The use of specific pathogens, such as viruses, bacteria, fungi, and nematodes, have been used as a pest management strategy to control insects of medical relevance [154]. Likewise, taking advantage of natural scorpion pathogens could also be an alternative to control their population. It has been reported that the filamentous fungus *Fusarium solani* can infect scorpions, resulting in deleterious effects such as movement and feeding reduction, leading ultimately to death [155]. As *F*. *solani* can also infect humans, its use may not be a safe approach for controlling scorpion populations, but understanding the mechanism by which it impairs scorpion’s movement may give hints for developing new strategies. Exoskeleton infections of *T*. *serrulatus* by *Candida* sp. and *Mucor* sp. have also been reported, causing behavioral changes similar to the ones reported for *F*. *solani* [156]. More recently, Brites-Neto and colleagues also sought for deleterious effects in *T*. *serrulatus* caused by fungi isolates [140]. An isolate identified as *Paecilomyces* sp. CMAA1686 showed interesting results. Two of the fungus’ secondary metabolites could kill scorpions, with lower undesirable repellency than other fungi and fractions, constituting a possible lead for the development of chemical control strategies.

Recently, many proposed pest management strategies relied on genetically modified individuals, or individuals infected with pathogens that impair reproduction or reduces offspring, introduced in hot spots with abundant pest population [157,158]. Through reproduction, these traits would be transmitted to the next generations, resulting in fewer individuals as offspring. The use of such approaches to control mosquito-borne diseases has achieved successful outcomes, specially using *Wolbachia*-infected *Aedes aegypti*, already being used in field trials around the world [159]. Despite their promising efficacy, genetic pest management, using gene drive and *Wolbachia* infection, has not been studied for containing scorpion infestations so far. As these techniques depend on mating between the modified and wild-type individuals, they would not be suitable to tackle *T*. *serrulatus* and *T*. *stigmurus* populations, as their reproduction is parthenogenetic.

The use of RNAi to silence essential genes has been considered as a good strategy for insect pest control, already tested in mites [160,161]. To employ this technique, knowledge of suitable target genes is paramount, as well as proper delivery systems [162]. As such knowledge concerning scorpions is very limited, this approach also has not been tested for controlling scorpion populations.

Several options for urban pest control have emerged in the past few years, mainly focusing on agents of vector-borne diseases, and are likely to shape the future of their related diseases [163–165]. Many of them could have been adapted to fight scorpion’s cities infestations, but, surprisingly, none of them has been even tested to this end. Taking advantage of this accumulated knowledge to adapt solutions for the scorpionism problem constitute a new avenue of research to tackle the current scorpion infestation in Brazil. However, to adequately pursue this goal, further studies on scorpion basic science concerning their ecology, biology, physiology, and genetics have also to be accomplished. Research in these fields should be encouraged in countries like Brazil, where scorpions are increasing as a health-concerning issue.

## 5. Concluding remarks

**Fig 6:**
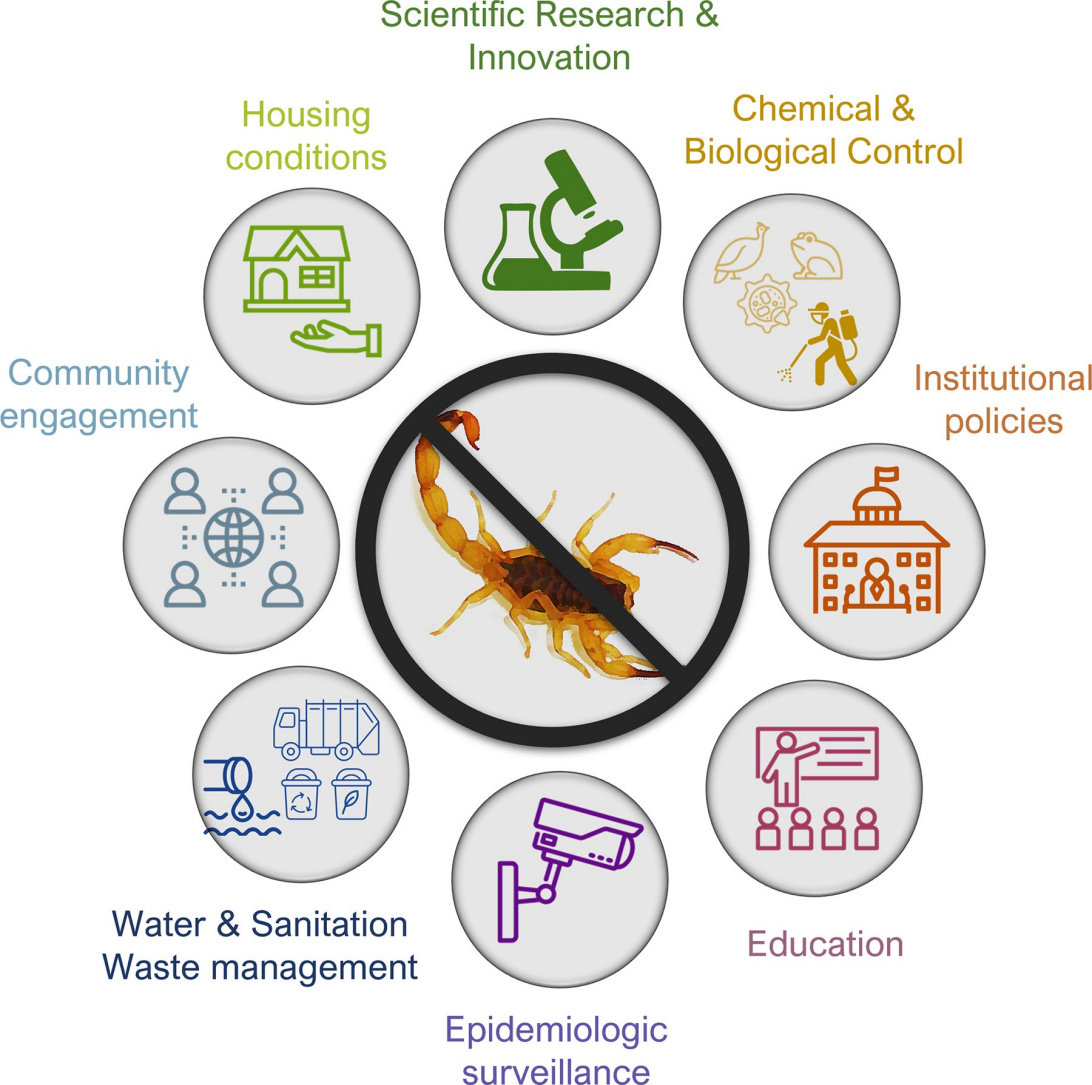
Different fields of measures that could help to contain the current scorpionism epidemic in Brazil. Icons used in this figure were obtained from and are currently available at the Noun Project (https://thenounproject.com/). Icons creator’s attributions: Sewer-BomSymbols TH; Surveillance -TozIcon TH; Pesticide Applicator—Luis Prado US; Toad–IconsProducer; Guinea Fowl—Georgiana Ionescu; Mould—nareerat jaikaew TH; Education and Politics—Nithinan Tatah; TH; Housing–Eucalyp; Research—romli ahmad SG; Society–vectorsmarket; Garbage recycling—Vectors Point PK.

The scientific knowledge on scorpions regarding their ecology [166–171], venom composition [172–175], envenomation pathophysiology [176–180], and treatment options [181] has much advanced in the past years. However, in this same period, the number of scorpion accidents and the geographical distribution of several noxious species has substantially increased in Brazil. This mismatch between research advances and the reality of scorpionism as a health problem reveals that the important efforts made by researchers are not being fully translated to solve this issue. Moreover, research fields important to contain scorpionism, such as accident prevention and pest control, have been disregarded and with no innovations presented (Fig 6).

As climate change and deforestation are rising and severely impacting Brazilian biomes [182], which seems to also affect scorpion fauna [37,38,40], we can foresee that scorpionism can have an even larger boom in the near future, if nothing is done to contain it. Therefore, this review aimed to call attention to these topics, expecting that the scientific community can come together with policy makers and the general population to avoid the situation to become even more critical than it already is.

## 6. Methods

This review was based in searches using the PubMed and Google Scholar engines, using as key words: “scorpionism,” “epidemiology,” “Brazil,” “*Tityus*,” “accident prevention,” “pest control,” in different combinations.

To assess the epidemiological data on scorpion accidents in Brazil, the SINAN (Sistema de Informação de Agravos de Notificação) database and IBGE (Instituto Brasileiro de Geografia e Estatística) data were consulted.

**Key Learning Points**

- Brazil is experiencing an explosion in the number of scorpion accidents in the past few years.
- Most of these scorpion accidents occur in the Southeast and Northeast region, but it has been notably expanding to other regions
- *Tityus serrulatus* is traditionally accountable for most of the scorpion accidents in different regions of Brazil, but other *Tityus* species deserve to be closely watched.
- Accident prevention and pest control measures can contribute to contain this problem, but no recent advances in these areas have been observed.

**Top Five Papers**

1. Lourenço WR. What do we know about some of the most conspicuous scorpion species of the genus *Tityus*? A historical approach. J Venom Anim Toxins Incl Trop Dis. 2015;21:20. doi: 10.1186/s40409-015-0016-9
2. Pimenta RJG, Brandão-Dias PFP, Leal HG, Carmo AO do, Oliveira-Mendes BBR de, Chávez-Olórtegui C, et al. Selected to survive and kill: *Tityus serrulatus*, the Brazilian yellow scorpion. PLoS ONE. 2019;14:e0214075. doi: 10.1371/journal.pone.0214075
3. Wen FH, Monteiro WM, Moura da Silva AM, Tambourgi DV, Mendonça da Silva I, Sampaio VS, et al. Snakebites and Scorpion Stings in the Brazilian Amazon: Identifying Research Priorities for a Largely Neglected Problem. Gutiérrez JM, editor. PLoS Negl Trop Dis. 2015;9:e0003701. doi: 10.1371/journal.pntd.0003701
4. Amado TF, Moura TA, Riul P, Lira AF de A, Badillo-Montaño R, Martinez PA. Vulnerable areas to accidents with scorpions in Brazil. Trop Med Int Health. 2021;26:591–601. doi: 10.1111/tmi.13561
5. Monteiro WM, Gomes J, Fé N, Mendonça da Silva I, Lacerda M, Alencar A, et al. Perspectives and recommendations towards evidence-based health care for scorpion sting envenoming in the Brazilian Amazon: A comprehensive review. Toxicon. 2019;169:68–80. doi: 10.1016/j.toxicon.2019.09.003

## Supporting information

S1 Table Table containing epidemiological data obtained from SINAN (Sistema de Informação de Agravos de Notificação) database from Brazilian Ministry of Health. Data related to the different aspects of reported scorpion accidents in Brazil are analyzed in the different table’s tabs.
(XLSX)
